# Deep learning assistance increases the detection sensitivity of radiologists for secondary intracranial aneurysms in subarachnoid hemorrhage

**DOI:** 10.1007/s00234-021-02697-9

**Published:** 2021-04-10

**Authors:** Lenhard Pennig, Ulrike Cornelia Isabel Hoyer, Alexandra Krauskopf, Rahil Shahzad, Stephanie T. Jünger, Frank Thiele, Kai Roman Laukamp, Jan-Peter Grunz, Michael Perkuhn, Marc Schlamann, Christoph Kabbasch, Jan Borggrefe, Lukas Goertz

**Affiliations:** 1 Institute for Diagnostic and Interventional Radiology, Faculty of Medicine and University Hospital Cologne, University of Cologne, Kerpener Straße 62, 50937 Cologne, Germany; 2 Department of Diagnostic and Interventional Radiology, University Hospital Düsseldorf, Düsseldorf, Germany; 3 Innovative Technologies, Philips Healthcare, Aachen, Germany; 4 Center for Neurosurgery, Department of General Neurosurgery, University of Cologne, Faculty of Medicine and University Hospital, University of Cologne, Cologne, Germany; 5 Department of Diagnostic and Interventional Radiology, University Hospital Würzburg, Würzburg, Germany; 6 Department of Radiology, Neuroradiology and Nuclear Medicine, Johannes Wesling University Hospital, Ruhr University Bochum, Bochum, Germany

**Keywords:** Aneurysms, Aneurysmal subarachnoid hemorrhage, CT angiography, Deep learning, Convolutional neural networks

## Abstract

**Purpose:**

To evaluate whether a deep learning model (DLM) could increase the detection sensitivity of radiologists for intracranial aneurysms on CT angiography (CTA) in aneurysmal subarachnoid hemorrhage (aSAH).

**Methods:**

Three different DLMs were trained on CTA datasets of 68 aSAH patients with 79 aneurysms with their outputs being combined applying ensemble learning (DLM-Ens). The DLM-Ens was evaluated on an independent test set of 104 aSAH patients with 126 aneuryms (mean volume 129.2 ± 185.4 mm^3^, 13.0% at the posterior circulation), which were determined by two radiologists and one neurosurgeon in consensus using CTA and digital subtraction angiography scans. CTA scans of the test set were then presented to three blinded radiologists (reader 1: 13, reader 2: 4, and reader 3: 3 years of experience in diagnostic neuroradiology), who assessed them individually for aneurysms. Detection sensitivities for aneurysms of the readers with and without the assistance of the DLM were compared.

**Results:**

In the test set, the detection sensitivity of the DLM-Ens (85.7%) was comparable to the radiologists (reader 1: 91.2%, reader 2: 86.5%, and reader 3: 86.5%; Fleiss κ of 0.502). DLM-assistance significantly increased the detection sensitivity (reader 1: 97.6%, reader 2: 97.6%,and reader 3: 96.0%; overall P=.024; Fleiss κ of 0.878), especially for secondary aneurysms (88.2% of the additional aneurysms provided by the DLM).

**Conclusion:**

Deep learning significantly improved the detection sensitivity of radiologists for aneurysms in aSAH, especially for secondary aneurysms. It therefore represents a valuable adjunct for physicians to establish an accurate diagnosis in order to optimize patient treatment.

## Introduction

Aneurysmal subarachnoid hemorrhage (aSAH) is caused by spontaneous rupture of an intracranial aneurysm and accounts for approximately 85% of non-traumatic SAH [1, 2]. With mortality ranging between 23% and 67%, aSAH poses a potentially life-threatening condition and a worldwide health burden leaving approximately 20% of long-term survivors permanently disabled [1, 3].

Accurate and reliable detection of a potentially ruptured intracranial aneurysm (RIA) is essential for diagnosis and the subsequent treatment concept in patients with non-traumatic SAH [4, 5]. In particular, timely aneurysm embolization by surgical or endovascular means is mandatory in order to prevent rebleeding, which may decisively affect patients’ prognosis. Generally, CT angiography (CTA) represents the imaging modality of choice to screen for aneurysms and is performed immediately upon radiological proof of SAH. The detection sensitivity of CTA for intracranial aneurysms is reported to range between 85% and 98% when compared to digital subtraction angiography (DSA) [6–8].

Due to an increasing workload of radiology departments, physician fatigue and the “satisfaction of search” phenomenon represent a real concern, aggravating the risk of missing relevant findings [9, 10]. Given that aneurysm detection on CTA proves to be challenging with misdiagnosis of aSAH eventually resulting in a poor clinical outcome, automated detection of intracranial aneurysms may be of valuable assistance to physicians [11–14]. Over the last decade, deep learning models (DLMs), in particular convolutional neural networks (CNNs), have shown great potential in performing diagnostic and analyzing tasks on medical imaging for different subspecialties [15–18, 19].

Previous studies have introduced several approaches for deep learning-based detection of aneurysms on CTA [18, 20–22] or magnetic resonance angiography (MRA) [23–25], compared the accuracy of the DLM to human readers, and investigated the deep learning-augmented diagnostic performance of physicians [20, 25]. However, these studies did not include patients with aSAH and focused on unruptured intracranial aneurysms (UIAs) [20, 25]. Therefore, if deep learning would enhance the diagnostic sensitivity of human readers of different experience levels for aneurysms in aSAH remains a relevant question. In a recent study, a DLM was introduced, which provided a high detection sensitivity of aneurysms in aSAH independent of cerebral circulation and bleeding severity [26].

The objective of the study was to investigate whether this DLM could increase the detection sensitivity of radiologists for intracranial aneurysms on CTA in patients with aSAH.

## Materials and methods

The institutional review board approved this retrospective, single-center study (reference number: 19-1329) and waived the necessity for written informed patient consent.

### Patient population and data collection

Two hundred ten consecutive patients with aSAH treated between January 2013 and December 2017 at our tertiary care university hospital were reviewed by the authors. Patients were excluded given the following criteria: (I) unavailable CTA scans (*n* = 16), (II) no aneurysm finding on CTA (*n* = 6), (III) motion artifacts on CTA (*n* = 2), (IV) insufficient contrast of CTA (*n* = 7), (V) preprocessing failure (*n* = 5), and (VI) previously treated aneurysms (*n* = 2).

Included CTA scans of patients treated between 2016 and 2017 served as the training set for the DLM (68 patients/79 aneurysms), whereas patients treated between 2013 and 2015 formed the test set (104 patients/126 aneurysms). These datasets were previously used for the training of the DLM and formed a part of the test set in the aforementioned study [26]. In this study [26], patients between 2010 and 2015 were used as a test set (in total: 185 patients with 215 aneurysms).

The included 170 scans of the present study were acquired at a single center on different multidetector CTs (iCT (*n* = 161), brilliance 64 (*n* = 3), and brilliance 16 (*n* = 6); *Philips Healthcare, Best, the Netherlands*) using a standardized clinical protocol for head & neck or head imaging with slice thickness ranging between 0.62 to 1.25 mm. CTA scans of two patients were acquired at referring institutions. After anonymization, CTA source images were exported to IntelliSpace Discovery for segmentation (ISD*, v3.0.6, Philips Healthcare*).

### Reference standard

In order to establish the aneurysm count and reference standard, a radiologist with 3 (L.P.), a neurosurgeon with 4 (L.G.), and a board-certified neuroradiologist with 12 years of experience (C.K.) in neurovascular imaging performed a double review of CTA and DSA (available in 153 patients, 89.0%) scans as well as their reports. Discrepancies were resolved in consensus.

The aneurysm findings were manually segmented by the aforementioned neurosurgeon and radiologist on ISD. Segmentation was performed interactively using a two-step, semiautomatic approach. First, a rough segmentation of the aneurysm was obtained by 3D voxel-wise regional thresholding. Afterwards, the segmentation was further edited manually using 2D editing tools. Additionally, the abovementioned readers collectively reviewed non-enhanced CT scans to determine respective Fisher grade of aSAH in consensus.

### Image preprocessing

In order to enable the DLM-based automatic aneurysm segmentation workflow, a preprocessing pipeline was employed [26]: First, a brain extraction algorithm was developed using Statistical Parametric Mapping software package version 8 (SPM8; *Wellcome Trust Centre for Neuroimaging*) to compute the brain mask [27]. Second, image standardization was performed by resampling to an isotropic resolution of 0.5 mm^3^ and intensity normalized. Third, a multi-scale vessel enhancement filter (consisting of two vessel-enhanced images, one with scale 0.5–5 voxels and the other with scale 5–15 voxels) was applied to the brain masked images enabling enhancement of the arteries from the background of the scans to distinguish between blood vessels and aneurysms [28]. Fourth, the original CTA image was normalized between 5 and 95% percentiles, while the vessel enhanced images were Z-score normalized.

### Deep learning model

The specifics of the used DLM and its training are described elsewhere [26]. In brief, 3D CNNs based on DeepMedic (*Biomedical Image Analysis Group, Department of Computing, Imperial College London*) [29] were used in this study.

Three separate DLMs (DLM-Orig, DLM-Vess, and DLM-LDim), which receive different inputs of the CTA datasets, were trained on the training set applying fivefold cross validation using an 80–20% training-validation split. By combining the outputs of the three DLMs, an ensemble model similar to the work of Kamnitsas et al. was created [30]. We refer to this combination strategy as DLM-Ens. To this end, the three trained DLMs were applied to the test set with every trained DLM consisting of five individual submodels that resulted from the fivefold cross-validation training approach. Using simultaneous truth and performance level estimation (STAPLE), outputs from these five submodels were fused together. Subsequently, the STAPLE outputs from the three DLMs were passed to the DLM-Ens to produce the final, fully automated three-dimensional aneurysm segmentations [31].

**Fig. 1:**
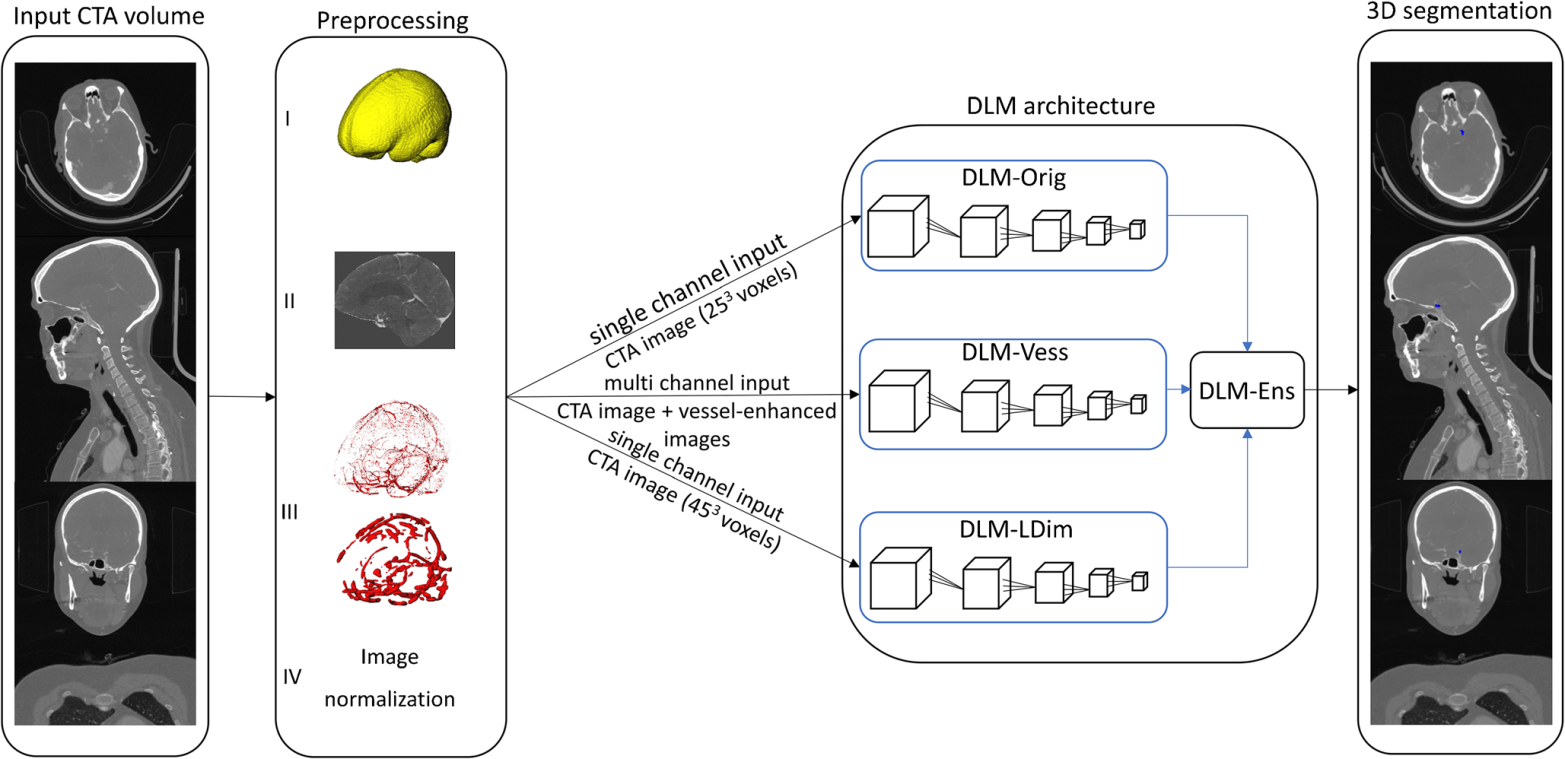
Automated workflow of image preprocessing (I: brain extraction, II: image standardization, III: vessel enhancement using two-vessel-enhanced images with scales of 0.5–5 voxels (superior) and of 5–15 voxels (inferior), and IV: image normalization), inputs to the deep learning models (DLMs), and model ensembling. In blue, the final 3D segmentation of an aneurysm of the left internal carotid artery. *CTA*, CT angiography

**Fig. 2:**
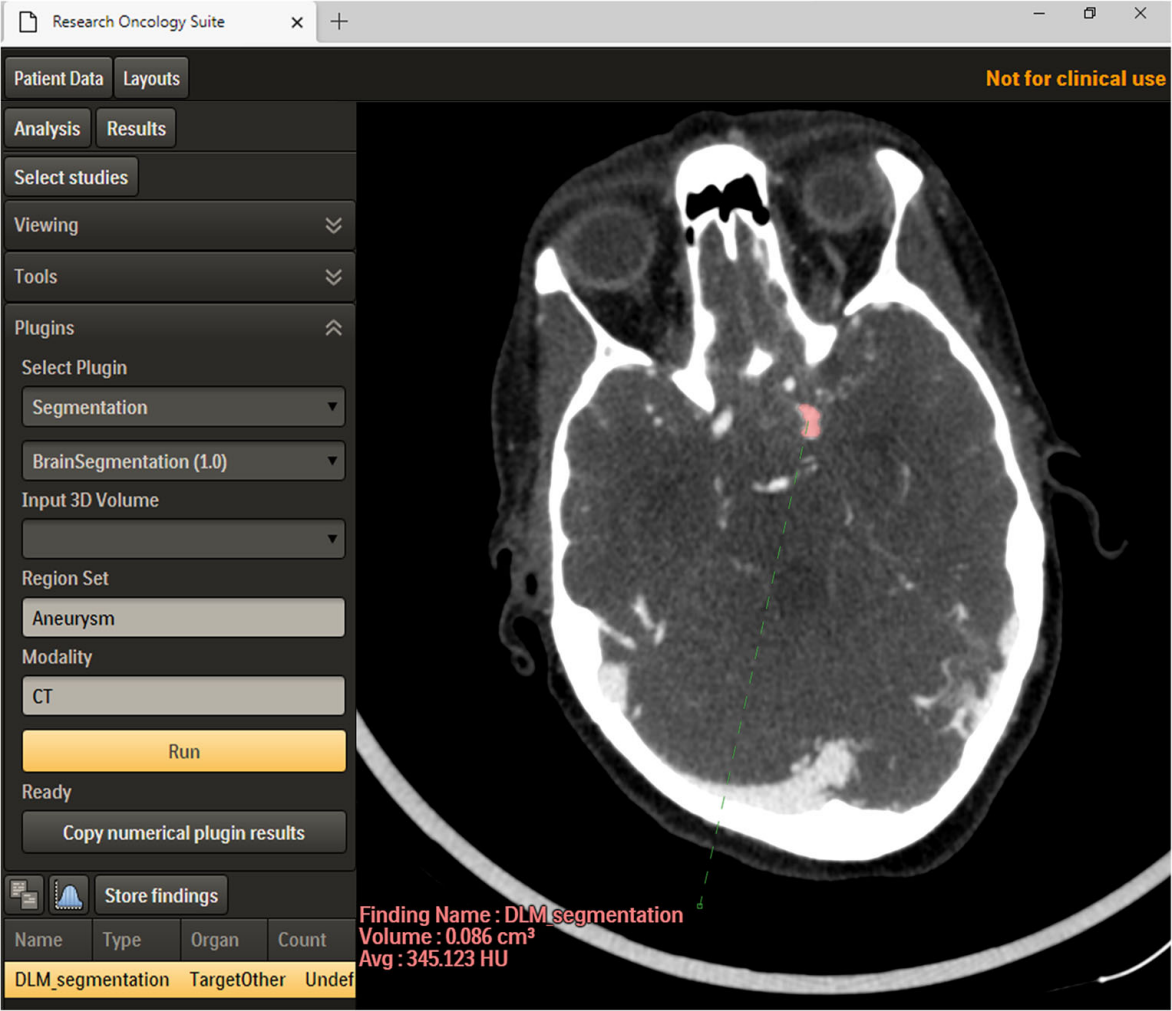
Browser-based fully automated aneurysm segmentation on IntelliSpace Discovery using the deep learning model

The fully automated workflow of image preprocessing and aneurysm detection is depicted in Fig. 1. The time needed for the DLM to fully automatically segment the aneurysms is about 3 min (including image preprocessing and model ensembling). Figure 2 displays the fully automated aneurysm segmentation on the IntelliSpace Discovery user interface.

### Detection of aneurysms by the radiologists

Anonymized and unlabeled CTA scans of the test set were presented to three radiologists with different levels of expertise (reader 1: 13 years (J.B.), reader 2: 4 years (U.H.), and reader 3: 3 years (A.K.) of experience in diagnostic neuroradiology) in random order for individual assessment of intracranial aneurysms. These readers were not involved in the definition of the reference standard. In order to simulate an emergency setting, which is the case during admission of a patient with aSAH, readers were instructed to perform their readings in a thorough but timely fashion. All readings were performed on the same IMPAX EE (*Agfa HealthCare N.V., Mortsel, Belgium*) workstation as used in clinical routine, applying the Multiplanar-Reconstruction-(MPR) tool if needed.

Readers were instructed that every dataset comprised at least one aneurysm and advised to report their findings in a Microsoft Excel (2016, *Microsoft Corp., Albuquerque, New Mexico, USA*) datasheet using the following labels: ICA (internal carotid artery), ACA (anterior cerebral artery including anterior communicating artery), MCA (middle cerebral artery), and POSTERIOR (posterior cerebral artery, posterior communicating artery, and vertebrobasilar territory) with respective vessel side attached. Beyond that, readers were blinded to patient and clinical data. Additionally, readers were instructed to record the reading time for each scan in seconds. After the initial reading, the readers were provided with the results from the DLM and could amend their readings accordingly in a separate column of the datasheet. The detection performance of the DLM-Ens and the radiologists were compared to the reference standard.

In order to investigate if the DLM may enhance the detection rate of the radiologists, results of readers 1, 2, and 3 were combined with the detections provided by the DLM.

### Statistical analysis

Statistical analysis was performed using Microsoft Excel and SPSS (*V22.0, IBM Corp., Armonk, NY, USA*), with *P* < 0.05 considered statistically significant. Categorical variables are presented as numbers and percentages. Continuous variables are reported as mean ± standard deviation (SD) and range. The sensitivity of the radiologists and of the DLM-Ens was calculated by comparison to the reference standard as provided by segmentations of aneurysms on ISD. Detection rates were compared using the Fishers’ exact test or the paired Student’s *t*-test, when appropriate. Interrater agreement was assessed using Fleiss κ with 0 indicating no, 0.01–0.2 slight, 0.21–0.40 fair, 0.41–0.6 moderate, 0.61–0.80 substantial, and 0.81–1.00 almost perfect agreement [32].

## Results

### Patient and aneurysm characteristics

In the test set, 126 aneurysms of 104 patients (mean age: 55.4 ± 14.3 years, 66 females) were segmented on ISD and comprised the reference standard. Baseline patient and aneurysm characteristics are outlined in Table 1. The majority of patients presented with a World Federation of Neurosurgical Societies (WFNS) score of 5 (32.7 %) and a Fisher grade 4 bleeding (53.8 %). Eighteen patients (17.3%) had multiple aneurysms. Manual segmentations yielded a mean aneurysm volume of 129.2 ± 185.4 mm^3^ (5.0–920.71 mm^3^). Aneurysms were located at the ACA in 39.7%, the MCA in 31.0%, the ICA in 16.7%, and the posterior circulation in 12.7%.

**Table 1:** Patient and aneurysm characteristics of the test set in absolute and relative values

Parameter	Value
Number of patients	104
Patient age (years; mean ± SD)	55.4 ± 14.3
Patients with multiple aneurysms	18 (17.3%)
Sex	
Female	66 (63.5%)
Male	38 (36.5%)
WFNS score	
1	29 (27.9%)
2	10 (9.6%)
3	14 (13.5%)
4	17 (16.3%)
5	34 (32.7%)
Fisher grade	
1	0 (0%)
2	5 (4.8%)
3	43 (41.3%)
4	56 (53.8%)
Total number of aneurysms	126
Aneurysm location	
Anterior circulation	110 (87.3%)
Internal carotid artery	21 (16.7%)
Anterior cerebral artery	50 (39.7%)
Middle cerebral artery	39 (31.0%)
Posterior circulation	16 (12.7%)
Aneurysm volume (mm^3^; mean ± SD)	129.2 ± 185.4
< 100 mm^3^	87 (69.0%)
> 100 mm^3^	39 (31.0%)

*WFNS*, World Federation of Neurosurgical Societies; *SD*, standard deviation

### Detection sensitivity of the DLM and of the radiologists

The DLM-Ens detected 108 of 126 aneurysms (sensitivity: 85.7%) while 87 false-positives findings were noted, corresponding to an average number of 0.84 false positives per scan. Missed aneurysms were predominantly located at the anterior circulation (72.2%) and had an average volume of 49.0 ± 43.1 mm^3^ (based on manual segmentations).

Reader 1, the neuroradiologist with 13 years of experience, detected 115 aneurysms correctly (sensitivity: 91.1%), reader 2 detected 109 (86.5%), and reader 3 detected 109 (86.5%). The mean sensitivity of the readers was 88.1%. The mean reading time of all three readers was 43.0 ± 14.12 s with reader 2 showing the longest reading time (45.8 ± 17.7 s; *P* = 0.017). Interrater agreement among the radiologists was moderate (Fleiss κ of 0.502; 95% confidence interval (CI): 0.416–0.588).

Table 2 provides detailed results regarding the missed aneurysms by the readers and the DLM. In particular, there were three aneurysms that were missed both by the DLM and the radiologists (Fig. 5): A mycotic aneurysm of the left anterior cerebral artery was probably missed due to its atypical, peripheral location. Two further aneurysms at the anterior communicating artery and the right posterior communicating artery were missed, probably due to their small size and because they were overlooked or misinterpreted as infundibula by the readers. For all readers combined, there were a total of 10 primary aneurysms, which were missed bythe readers (average volume: 82.0 ± 60.8 mm^3^, Table 2). When excluding the above-mentioned relatively large mycotic aneurysm of the left ACA (n=3), these findings showed a volume of 40.9 ± 21.7 mm^3^ and were located at the anterior (n=3) and posterior (n=4) communicating arteries.

**Table 2:** Aneurysms missed by the readers and the DLM, deep learning model. *SD*, standard deviation

	Reader 1	Reader 2	Reader 3	DLM
Missed aneurysms	11	17	17	18
Thereof missed secondary aneurysms, %	10 (90.9)	13 (76.5)	12 (70.6)	3 (16.6)
Volume (mm^3^; mean ± SD)	83.7 ± 69.4	55.5 ± 64.4	49.7 ± 47.0	49.0 ± 43.1
Anterior circulation, %	7 (63.6)	12 (70.6)	10 (58.8)	13 (72.2)
Posterior circulation, %	4 (36.4)	5 (29.4)	5 (29.4)	5 (27.8)

### DLM-augmented detection sensitivity of the radiologists

After disclosure of the DLM results, reader 1 found 8 additional aneurysms (thereof 8 secondary aneurysms), reader 2 found 14 (13 secondary aneurysms), and reader 3 found 12 (9 secondary aneurysms) that they missed before. For all readers combined, 88.2% of additionalaneurysms were secondary aneurysms. Consequently, detection sensitivity improved to 97.6%, 97.6%, and 96.0% for readers 1, 2, and 3 with a mean overall sensitivity of 97.1% (*P* = 0.024). Furthermore, interrater agreement increased to almost perfect with a Fleiss κ of 0.878 (95% CI: 0.788–0.969). Table 3 provides detailed results regarding aneurysm detection by radiologists alone and in combination with the DLM as well as their reading time.

**Table 3:** Sensitivity of the three radiologists alone and combined with deep learning-generated detections and their individual reading times

	Sensitivity, in % (detected/overall)			Reading time
	Without DLM	With DLM	Difference	(s; mean ± SD)
Reader 1	91.3 (115/126)	97.6 (123/126)	6.3 (8/126)	40.5 ± 12.8
Reader 2	86.5 (109/126)	97.6 (123/126)	11.1 (14/126)	45.8 ± 17.7
Reader 3	86.5 (109/126)	96.0 (121/126)	9.5 (12/126)	42.7 ± 10.6
Mean	88.1	97.1	9.0	
95% CI	81.2–95.0	94.8–99.4	2.9–15.0	
*P*-value		0.024 (vs. without DLM)*		0.017

Reader 1: 13 years, reader 2: 4 years, and reader 3: 3 years of experience in diagnostic neuroradiology. *DLM*, deep learning model; *CI*, confidence interval; *SD*, standard deviation

*Determined by paired Student’s *t*-test

The DLM significantly enhanced the detection of the radiologists for aneurysms of both small (< 100 mm^3^) and large (> 100 mm^3^) volume as outlined in Table 4.

**Table 4:** Sensitivity of the three radiologists alone and combined with deep learning-generated detections for small and large aneurysms

	Sensitivity for aneurysms < 100 mm^3^, in % (detected/overall)			Sensitivity for aneurysms > 100 mm^3^, in % (detected/overall)		
	Without DLM	With DLM	Difference	Without DLM	With DLM	Difference
Reader 1	92.0 (80/87)	97.7 (85/87)	5.7 (5/87)	89.7 (35/39)	97.4 (38/39)	7.7 (3/39)
Reader 2	83.9 (73/87)	97.7 (85/87)	13.8 (12/87)	92.3 (36/39)	97.4 (38/39)	5.1 (2/39)
Reader 3	83.9 (73/87)	95.4 (83/87)	11.5 (10/87)	92.3 (36/39)	97.4 (38/39)	5.1 (2/39)
Mean	86.6	96.9	10.3	91.4	97.4	6.0
95% CI	81.3–91.9	95.4–98.4	5.7–14.9	89.7–93.1	97.4–97.4	4.3–7.7
*P*-value		0.024 (vs. without DLM)*			0.020 (vs. without DLM)*	

Reader 1: 13 years, reader 2: 4 years, and reader 3: 3 years of experience in diagnostic neuroradiology. *DLM*, deep learning model; *CI*, confidence interval; *SD*, standard deviation

*Determined by paired Student’s *t*-test

**Fig. 3:**
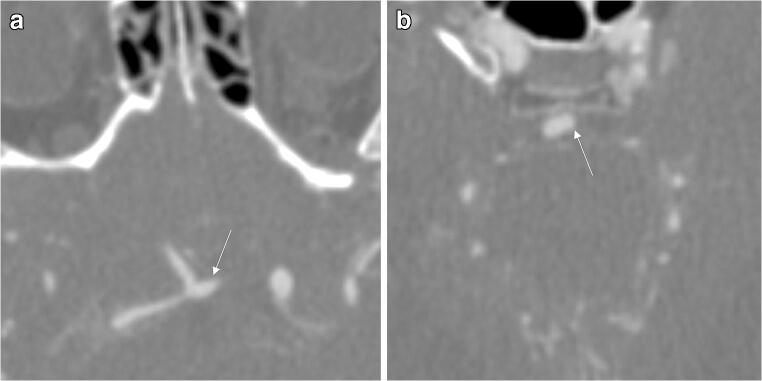
Axial CT angiography source images of a 77-year-old male with aneurysmal subarachnoid hemorrhage (Fisher 2). All three readers detected the aneurysm of the anterior communicating artery (**a**, arrow) but missed the aneurysm of the basilar head (**b**, arrow). The deep learning model detected both aneurysms

**Fig. 4:**
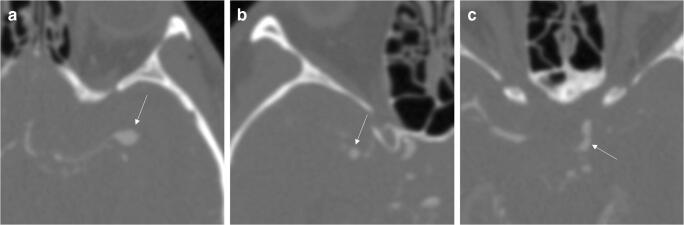
Axial CT angiography source images of a 55-year-old male with aneurysmal subarachnoid hemorrhage (Fisher 4) and aneurysms (arrows) of the left middle cerebral artery (**a**), the right middle cerebral artery (**b**), and the left internal carotid artery (**c**). Reader 1 missed the aneurysm of the right middle cerebral artery while readers 2 and 3 missed the aneurysm of the left internal carotid artery. The deep learning model detected all aneurysms

**Fig. 5:**
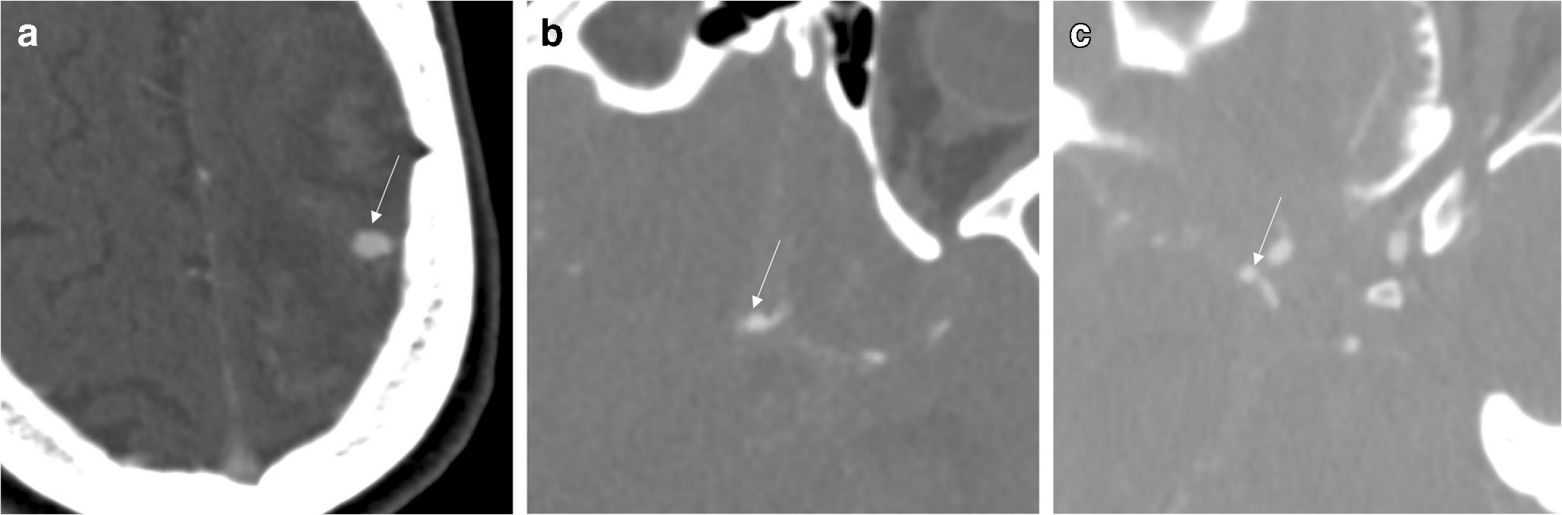
Axial CT angiography source images of the three aneurysms, which were missed by the readers and the deep learning model. **a** A mycotic aneurysm of the left anterior cerebral artery (arrow) in a 69-year-old male with aneurysmal subarachnoid hemorrhage (Fisher 4). **b** Aneurysm of the anterior communicating artery (arrow) in a 41-year-old female with aneurysmal subarachnoid hemorrhage (Fisher 4). **c** Aneurysm of the right posterior communicating artery (arrow) in a 33-year-old female with aneurysmal subarachnoid hemorrhage (Fisher 3)

Exemplary aneurysm findings are provided in Figs. 3, 4, and 5.

## Discussion

In the present study, we aimed to investigate whether a DLM could increase the detection sensitivity of radiologists for intracranial aneurysms on CTA in patients with aSAH. Deep learning assistance significantly improved the detection sensitivity of the readers to more than 95% independent of the experience level, predominantly by increasing the detection rate for secondary aneurysms. The DLM could assist readers in the detection of both small and large aneurysms while leading to an increase of interrater agreement from moderate to almost perfect.

Previous studies have evaluated DLMs for the detection of intracranial aneurysms on CTA [18, 20–22] and time-of-flight (TOF)-MRA [23–25] and investigated whether deep learning enhancement could increase the diagnostic performance of human readers [20, 25]. In the study by Park et al., artificial intelligence assistance increased the detection sensitivity of radiologists and of a neurosurgeon (2–12 years of experience) for UIAs on CTA significantly from 83% to 89% [20]. In a TOF-MRA study by Faron et al., deep learning assistance boosted the detection sensitivity of radiologists (2 and 12 years of experience) for UIAs, albeit without reaching statistical significance [25].

The present study is the first that investigated the performance of deep learning-assisted detection of aneurysms in patients with SAH by radiologists. The detection sensitivity of the DLM was 86%, which was comparable to that of readers in the present study (87%–92%) and to that of physicians for UIAs on CTA (83% for aneurysms > 3 mm [20]) and slightly lower to that of DLMs for UIAs on TOF-MRA (90% [24]). Given the accumulation of blood in the subarachnoid space in patients with aSAH, one might expect that the performance of the DLM may be impaired by the presence of hyperdense material adjacent to the hyperdense arteries. However, the DLM of the present study finds a considerably low number of false positives per scan (less than 1), which is unaffected by the degree of the hemorrhage [26]. This number of false-positive findings per scan is in fact lower than in other studies investigating deep learning-based detection of predominantly unruptured aneurysms on TOF-MRA (e.g., Sichtermann et al.: 6 [24], Ueda et al.: 10 [23]) or CTA (e.g., Dai et al.: 9 [22], Yang et al.: up to 14 [18]), which questions their feasibility of automated detection in clinical routine. The time needed for the DLM to fully automatically segment the aneurysms is about 3 min and therefore feasible in an emergency workflow, in which the inference can be performed after acquisition and integrated with the reconstruction process while transferring the data into the picture archiving and communication system.

Interestingly, the DLM and the readers missed a comparably large mycotic aneurysm at a peripheral branch of the anterior cerebral artery. Regarding the DLM, it might be speculated that a more elaborate training including atypical aneurysm locations would further increase the detection sensitivity. Based on these considerations, a large-volume, multi-center registry training study should be performed prior to implementation of the DLM into clinical practice.

Alongside the aforementioned studies, the DLM boosted the detection sensitivity of all three radiologists with different experience levels of neurovascular imaging for small and large aneurysms, even for reader 1 with 13 years of experience [20, 25]. In the study by Park et al. (which also included CTA scans without aneurysms), artificial intelligence significantly increased the sensitivity of human readers from 83% to 89% [20], whereas, in the present study, the DLM allowed to increase the sensitivity to a larger extend (from 88% to 97%). In the TOF-MRA study by Faron et al., deep learning assistance resulted in a similar detection sensitivity (98% and 97%, respectively). However, the detection sensitivity of human readers alone was already 94% and 95% [25]; hence, the DLM did not increase human sensitivity to the same extent as in the present study.

Given the complexity of intracranial vessels, CTA-based detection of aneurysms proves to be time-consuming and challenging, therefore showing a large variability among physicians, even for experienced readers and especially for small aneurysms [11, 12, 14]. Consequently, only a moderate interrater agreement for the three readers was noted with the highly experienced reader achieving a higher detection rate than the less experienced ones, as could be expected. After combining their detections with deep learning-generated findings, an almost perfect interrater agreement was noted with the DLM providing additional findings, including aneurysms < 100 mm^3^ (which translates to a maximum diameter of 6 mm). Therefore, augmenting physicians’ performance could potentially lead to a more precise and consistent interpretation of imaging data. Of note, the DLM applied in this study had a higher impact regarding the increase of interrater agreement compared to the study by Park et al. (0.376 vs. 0.060) [20].

Given the increasing workload of radiology departments, DLMs and computer-aided detection algorithms represent a valuable assistance to clinicians to deal with the growing amount of data that need to be analyzed [16, 33, 34]. With an overall detection rate of 86%, the DLM itself was inferior to a trained and experienced neuroradiologist, who had a detection rate of 91%. However, the neuroradiologist missed 11 additional aneurysms, which can be most likely attributed to the “satisfaction of search” phenomenon. The DLM detected 8 of these 11 aneurysms. Hence, deep learning-generated detections provide an important adjunct in an emergency setting, especially for unexperienced and potentially overstrained readers, for which the DLM detected 14 and 12 additional aneurysms, respectively. Of note, a total of 10 primary aneurysms were missed by all readers combined, the majority (n=9) by the unexperienced readers. Despite the knowledge that every dataset harbored at least one aneurysm, these findings were most likely missed due to their atypical, peripheral localization and their small size at the anterior and posterior communicating artery, consequently leading to an oversight because they were misinterpreted as infundibula.

The “satisfaction of search” phenomenon poses a relevant concern in radiology and medicine in general [9, 10]. In the current study, the radiologists were instructed that every dataset contained at least one aneurysm; consequently, readers were able to detect at least one aneurysm in most cases. It is likely that these prerequisites have artificially increased the overall detection sensitivity of the radiologists. However, in clinical practice, the radiologist would also expect to detect an aneurysm after the diagnosis of basal SAH, even in the comparatively rare case of non-aneurysmal SAH. Despite this advantage for the radiologists, the DLM achieved comparable results to the readers and significantly increased their detection sensitivity. The effects might therefore be even stronger in clinical routine. Furthermore, deep learning assistance especially helped the radiologists to detect secondary aneurysms, which are present in about 20% of patients in aSAH (17% in the present study) [35, 36]. Eighty-eight percent of the additional aneurysms detected by the DLM were in fact secondary aneurysms. These results demonstrate that the DLM provides relevant support to human readers, in particular to physicians that are impaired by the satisfaction of search and lack of training or lack concentration due to fatigue.

## Limitations

The following limitations need to be considered: First, the physiological distress of an emergency setting like an aSAH situation cannot be fully reproduced in a retrospective study. Second, the retrospective collection of the included data will have led to a relative spectrum bias. Third, the readers were aware that the patients in the test set harbored at least one aneurysm. Therefore, the only unknown variable was the number of patients who had secondary aneurysms. This prerequisite of the study design most likely led to an artificially increased sensitivity of the radiologists. Fourth, given that CTA scans of patients with non-aneurysmal SAH were not included, there is no control group of patients without aneurysms, which does not allow for the evaluation of a specificity. Fifth, the included CTA datasets in this study were almost exclusively acquired at a single institution with a fixed scanning protocol and of sufficient image quality, which does not reflect daily clinical practice and limits the generalizability of the algorithm. Hence, the findings and the true clinical impact of this study need to be confirmed in a prospective, multi-center setting, which allows for external validation and in which deep learning-generated detections are implemented directly in the clinical workflow.

## Conclusions

In patients with aSAH, the DLM significantly increased the detection rate of radiologists for intracranial aneurysms to more than 95%. Additional findings were predominantly secondary aneurysms. These results indicate that the integration of deep learning assistance could provide a valuable adjunct to radiologists for accurate aneurysm detection among aSAH patients.

## Data Availability

The datasets generated/analyzed during this study are not publicly available due to data protection but are available from the corresponding author upon reasonable request.
